# Synergistic Analysis of Protein Corona and Haemoglobin Levels Detects Pancreatic Cancer

**DOI:** 10.3390/cancers13010093

**Published:** 2020-12-30

**Authors:** Damiano Caputo, Luca Digiacomo, Chiara Cascone, Daniela Pozzi, Sara Palchetti, Riccardo Di Santo, Erica Quagliarini, Roberto Coppola, Morteza Mahmoudi, Giulio Caracciolo

**Affiliations:** 1 Department of Surgery, University Campus Bio-Medico di Roma, Via Alvaro del Portillo 200, 00128 Rome, Italy; d.caputo@unicampus.it (D.C.); c.cascone@unicampus.it (C.C.); r.coppola@unicampus.it (R.C.); 2 Department of Molecular Medicine, Sapienza University of Rome, Viale Regina Elena 291, 00161 Rome, Italy; luca.digiacomo@uniroma1.it (L.D.); daniela.pozzi@uniroma1.it (D.P.); sara.palchetti@uniroma1.it (S.P.); riccardo.disanto@uniroma1.it (R.D.S.); 3 Department of Chemistry, Sapienza University of Rome, P.le Aldo Moro 5, 00185 Rome, Italy; erica.quagliarini@uniroma1.it; 4 Department of Radiology and Precision Health Program, Michigan State University, East Lansing, MI 48824, USA; mahmou22@msu.edu

**Keywords:** pancreatic cancer, graphene oxide, early detection

## Abstract

**Simple Summary:**

Pancreatic ductal adenocarcinoma (PDAC) is often diagnosed at an advanced stage and is burdened by poor prognosis. Its early diagnosis is rare because the disease is frequently asymptomatic for a long time. Our research aims to identify a useful and simple diagnostic tool for early PDAC detection. The combination of user-friendly, nanotechnology-based tools and standard blood tests showed high accuracy in discriminating PDAC patients from healthy subjects. If confirmed in large-cohort studies, these findings could represent an innovative and non-invasive method with a potential impact in clinical practice in early detection of PDAC.

**Abstract:**

Simultaneous detection of multiple analytes from a single biological sample is gaining more attention in the development of more reliable and point-of-care diagnostic devices. We developed a multiplexed strategy that combined outcomes of clinical biomarkers with analysis of the protein corona that forms around graphene oxide sheets upon exposure to patient’s plasma. As a paradigmatic case study, we selected pancreatic ductal adenocarcinoma (PDAC), mainly because of the absence of effective detection strategies that resulted in an extremely low five-year survival rate after diagnosis (<10%). Association of protein corona analysis and haemoglobin levels discriminated PDAC patients from healthy volunteers in up to 90% of cases. If further confirmed in larger-cohort studies, this approach may be used in the detection of PDAC.

## 1. Introduction

A recent paper comparing records of 164,000 people in 21 countries on five continents reported that cancer is overtaking heart disease as the largest killer in developed countries [1]. Among different cancer types, pancreatic ductal adenocarcinoma (PDAC) is on the rise [2]. PDAC-related deaths have already overtaken breast cancer and remain on track to pass colorectal cancer as the second leading cause of deaths in developed countries in the next decade.

PDAC’s biological behavior and advanced stage at the moment of diagnosis are the main factors resulting in a poor prognosis [3]. In the early stages, PDAC is often asymptomatic or associated with mild and non-specific symptoms (e.g., nausea and/or loss of appetite) that can be attributed to other diseases [4]. Severe symptoms (e.g., intense pain and/or jaundice) that lead to medical consultation are typically associated with the advanced stage of the disease [5]. Nevertheless, the increase of obesity and diabetes, two risk factors for PDAC, contribute to more diagnoses and deaths from this disease [6]. However, there is still a lack of screening programs that can be offered to subjects potentially at higher risk of PDAC such as diabetics, patients affected by intraductal papillary mucinous neoplasm (IPMN), or patients with specific genetic syndromes [7,8,9]. Consequently, less than 10% of diagnosed PDACs survive five years after the diagnosis. Five-year survival rates may increase up to 15–20% when radical surgical resection is performed; unfortunately, at the moment of diagnosis, surgery is precluded for 80% of patients, due to the local invasion of vascular structures or distant metastases [10].

Recent evidence has demonstrated that the time interval between cancer initiation and clinical manifestation is longer than 10 years [11]. This finding suggests a large window for early detection and explains the continuous search for valuable tools for early diagnosis of PDAC.

Over the last few years, extensive efforts have been aimed at identifying new PDAC biomarkers. Recent improvements in molecular technologies have enabled clinicians to better identify the onset and progression of PDAC through related altered/mutant genes, RNAs, proteins, lipids, carbohydrates, and small metabolites [12,13]. However, although the number of proposed novel biomarkers (e.g., mi-RNAs, methylation biomarkers, circulating tumor DNA, circulating tumor cells, and exosomes) has been increasing during the last few years, there is still no clear evidence of their efficacy since none have proven high sensitivity and specificity [14]. Nonetheless, based on the encouraging results obtained in the management of other cancers, the role of volatile organic compounds (VOCs) in exhaled breath has been tested for PDAC and demonstrated the ability to distinguish adenocarcinoma from non-cancers with 70% and 74% sensitivity and specificity, respectively [15].

Consequently, to date, despite its 80% sensitivity and 80% specificity (37 U/mL cut-off) [16], carbohydrate antigen 19-9 (Ca 19.9) remains the only marker the Food and Drug Administration (FDA) approved in clinical practice for detection of PDAC. Unfortunately, Ca 19.9 is more useful during patient follow-up than for screening and diagnosis [17]. Several of the above-cited biomarkers have also been proposed to improve the diagnostic ability of Ca 19.9 [16,18]. However, they still have not found real applicability in routine clinics as they do not fulfil the ASSURED (Affordable, Sensitive, Specific, User-friendly, Rapid and robust, Equipment-free, and Deliverable to end-users) criteria required by the World Health Organization (WHO) for procedures for cancer screening and detection.

It is increasingly being reported that simultaneous detection of different analytes from a single biological sample may result in more robust and accurate diagnostic objectives compared to a single biomarker [19]. This approach has recently been implemented by multiplexed point-of-care testing (xPOCT), which aims to obtain as much information as possible from a single sample of body fluid (e.g., blood, urine, saliva, or sweat). The information was then processed with minimal user manipulation and provided accurate results [20]. For example, a combination of blood levels of haemoglobin (Hb), albumin, lymphocyte, and platelet has emerged as an important prognostic factor for postoperative survival of PDAC patients [21], but its role in diagnosis has not been established so far.

Systemic inflammatory response biomarkers (SIRBs) such as white blood count (WBC), neutrophils to lymphocytes ratio (NLR), derived-NLR (d-NLR), and platelets to lymphocytes ratio (PLR) have also attracted considerable attention for the diagnosis and prognosis of different types of tumors, including PDAC [22]. These markers are often altered in neoplastic conditions and receive a lot of emphasis because of their easy dosage, calculation, and reproducibility with standard laboratory tests. Electrochemical immunosensors have been used to simultaneously measure the concentration of common tumor markers (i.e., AFP, ferritin, CEA, hCG-β, CA 15-3, CA 125, and CA 19-9) [23]. The most widely used multiplexed imaging technology combines data from computer tomography (CT) with the physiological imaging data of Positron Emission Tomography (PET), providing accurate, functional, and anatomical information [24]. However, despite considerable efforts and numerous publications, no multiplexed technology for PDAC detection has been developed so far.

To address this gap, we developed a multiplexed strategy that combined values of clinical biomarkers with characterization of the PDAC-specific protein and corona of graphene oxide (GO). This personalized protein layer [25,26] surrounds GO nanosheets after exposure to human plasma (HP) of PDAC patients. By combining blood levels of Hb with abundant low-molecular-weight proteins within the PDAC protein corona (20–30 kDa), we obtained an area under the curve (AUC) of 0.961. This test distinguished PDAC patients from healthy individuals with 82.4% sensitivity and 97.1% specificity overcoming the prediction ability of a single parameter. These results provide the basis for future developments of multiplexed testing for the early detection of PDAC.

## 2. Results

Size and zeta-potential experiments showed that pristine GO sheets exhibited a mean lateral size of about 700 ± 23 nm and zeta-potential of −32 ± 3 V. As a first step, GO sheets were exposed to HP of PDAC patients and healthy individuals, leading to protein corona formation. Next, coronas were isolated and characterized by 1-dimensional (1D) SDS-PAGE.

**Figure 1:**
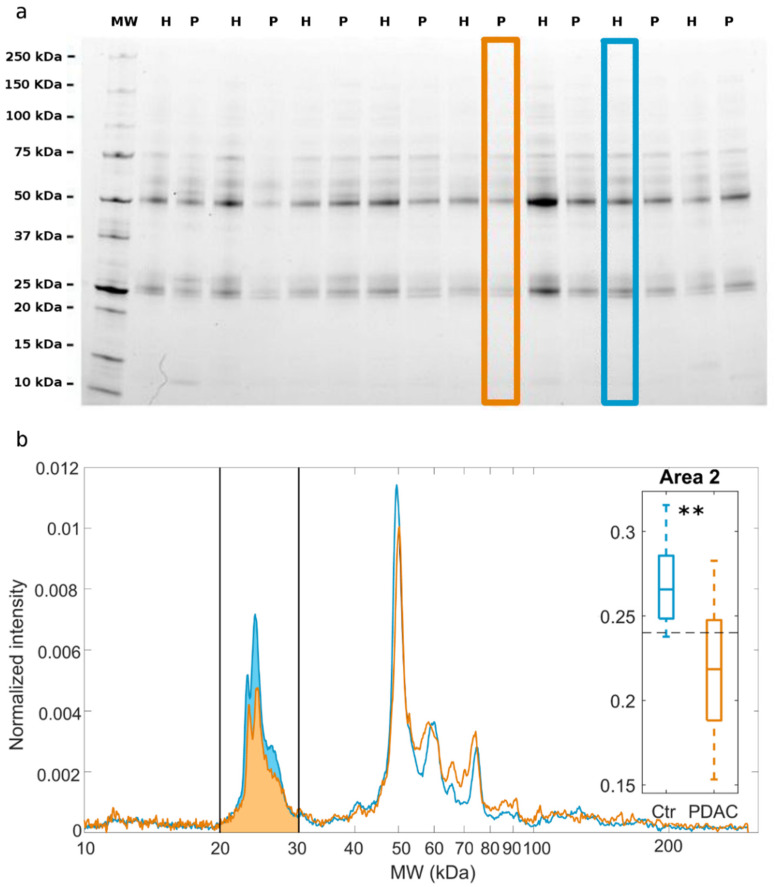
(**a**) Following exposure of graphene oxide (GO) sheets to human plasma, a protein corona forms. After protein corona isolation by centrifugation and protein corona analysis by 1D SDS-PAGE, an image of the gel is obtained. In the gel image, each lane refers to the protein profile from a single human subject, being either a pancreatic ductal adenocarcinoma (PDAC) patient (P) or a healthy volunteer (H). (**b**) 1-dimensional (1D) profiles were obtained by densitometric analysis of the two lanes that are indicated with orange (P) and blue (H) solid boxes in panel a. Black vertical solid lines identify a region of molecular weight between 20 and 30 kDa, hereafter indicated as Area 2, where the largest difference between the two 1D profiles is observed. A boxplot of the computed Area 2 for all the processed samples (34 PDAC vs. 34 healthy subjects) is reported in the inset. Whiskers span from the minimum to the maximum measured values, and boxes indicate the 25th–75th percentile range and horizontal segments represent the median values of the distributions. ** indicate a Student *p*-value < 0.001.

**Figure 2:**
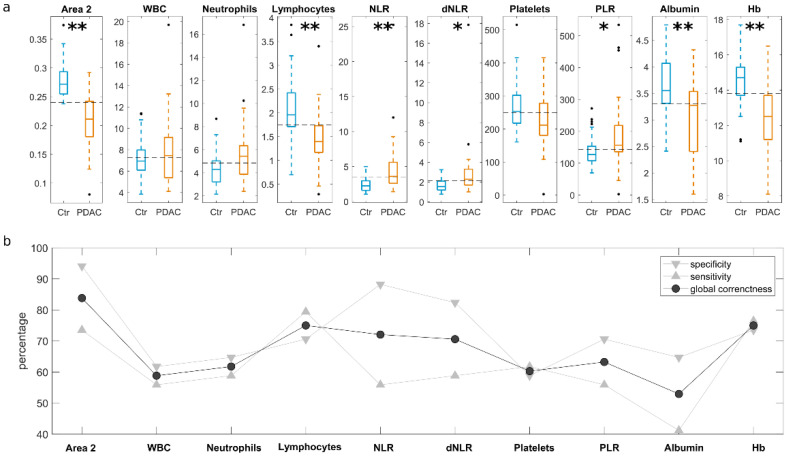
(**a**) Boxplots of electrophoretic and clinical parameters for PDAC patients (orange) and control (i.e., non-oncological) subjects (blue) sample distributions. Whiskers span from the minimum to the maximum measured values, and boxes indicate the 25th–75th percentile range and horizontal segments represent the median values of the distributions. Asterisks correspond to Student’s *t*-test *p*-values: * *p* < 0.05; ** *p* < 0.001. (**b**) Specificity, sensitivity and global correctness of the test’s discrimination ability for each of the investigated parameters.

Figure 1a shows a representative gel image where each lane reflects the protein pattern formed around GO sheets after interaction with HP from a single donor. In Figure 1b, we compared the average protein profiles from a PDAC patient (orange line) and from a healthy subject (cyan line) obtained by densitometric analysis of the gel lanes. The results suggest that the main difference in the protein profiles was in the molecular weight region within 20–30 kDa (hereafter indicated as “Area 2”). The boxplot in the inset summarize the experimental changes of Area 2 between PDAC and non-oncological distributions. The calculated *p*-value from Student’s *t*-test read a value below 10^−9^, thus indicating an extremely high statistical significance of the observed differences. This demonstrates that Area 2 alone would be a good classifier for PDAC with a positive predictive value of 83.8%. Next, we compared outcomes of standard laboratory tests and SIRBs between the two classes of donors (Figure 2a). We found out that the levels of lymphocytes (*p* = 0.0001), NLR (*p* = 0.001), dNLR (*p* = 0.013), PLR (*p* = 0.03), albumin *(p* = −0.0008), and Hb (*p* = 0.000002) were significantly altered in PDAC patients.

**Figure 3:**
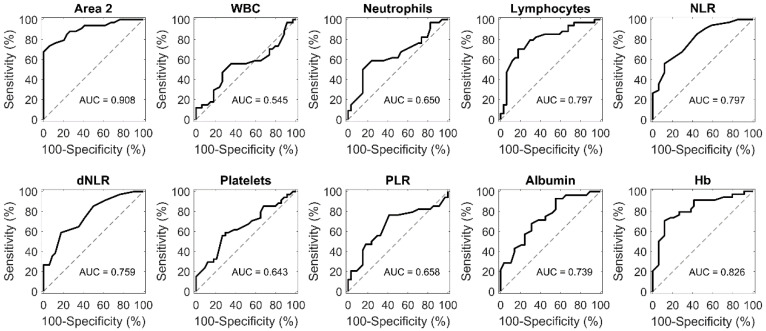
Receiving operative curve (ROC) curve and corresponding area under the curve (AUC) for each of the indicated parameters.

However, results reported in Figure 2b reveal that predictive values offered by laboratory tests and SIRBs were still lower than the positive predictive value of Area 2. This trend was confirmed by receiving operative curve (ROC) analysis and the corresponding AUC values, which were computed for all the employed parameters. As shown in Figure 3, Area 2 reached the largest AUC (0.91), followed by Hb (AUC = 0.83).

**Figure 4:**
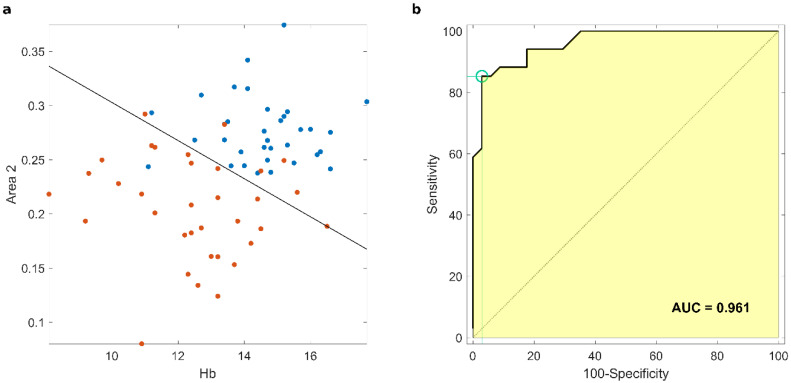
(**a**) Scatter plots for the best couple of PDAC predicting parameters, i.e., Area 2 and haemoglobin (Hb) concentration given in grams per deciliter. Each data point corresponds to a single sample (orange for PDAC, blue for non-oncological coronas), and solid black line depicts the output of the linear discriminant analysis for the two multivariate distributions. (**b**) AUC obtained by coupling Area 2 and Hb as classifiers.

As a next step, we monitored the combination of the outcomes of protein corona analysis and the clinical tests. Among 45 possible couplings, the combination of Area 2 with Hb levels (Figure 4a) returned the best values of global correctness (89.7%), sensitivity (82.4%), and specificity (97.1%) (Figure S1). Moreover, we obtained an area under the curve (AUC) of 0.961 (Figure 4b). The application of the test on healthy subjects and patients with early-stage pancreatic cancer (stage I and II) provided similar output values of sensitivity and specificity (86.7% and 95.8%, respectively) (Figure S2).

## 3. Discussion

Early diagnosis of PDAC is a significant unmet clinical need that can potentially save many lives. Recent advances in nanoparticle (NP) research have raised new hopes in the development of cancer diagnostic techniques. When NPs are exposed to human fluids, they are covered by a protein corona whose composition is disease-specific, i.e., it can be altered in people with disorders but not in healthy subjects. Recently, much effort has been devoted to exploiting the NP-protein corona for early detection of cancer [27,28]. Potential biomarkers are analyzed within “corona proteins” by liquid chromatography (LC) in tandem with mass spectrometry (MS/MS). However, biomarker discovery by LC-MS/MS poses serious concerns about sustainability on a larger scale. In particular, the absence of a clear relationship between elevation of the concentration of such biomarkers in plasma and at the surface of NPs, and the poor alignment to the ASSURED criteria stated by WHO for cancer screening and detection, is concerning.

Among benchtop techniques, SDS-PAGE is a straightforward method to investigate protein corona (PC) composition. In previous works, global information about PC composition provided by SDS-PAGE has successfully distinguished different classes of donors (e.g., cancer vs. non-cancer patients) [29,30]. This approach was used in this study to compare the PC that formed around GO sheets following exposure to HP from PDAC patients and healthy volunteers. Our findings suggest that the protein patterns from PDACs were significantly less enriched with proteins in the molecular weight range between 20 and 30 kDa. In this molecular weight range, apolipoprotein A1 (APOA1) was recently found to be the most enriched plasma protein in the GO corona [29]. Furthermore, it is known that apolipoprotein A1 has a high affinity to the graphene and GO surface [31,32], and it is recognized as a potential biomarker for PDAC [33,34,35].

Simultaneous measurement of different substances from a single body fluid (e.g., blood and/or urine) has recently become more critical for in vitro diagnostic approaches [19]. Here, we evaluated the blood levels of several clinical markers in their ability to discriminate PDAC patients from healthy subjects, and they were ranked in the following order: Hb > Lymphocytes > NLR > dNLR > Albumin > PLR > Neutrophils > Platelets > WBC. The decreased levels of Hb and albumin in the presence of PDAC can be explained by the impact of the tumor’s presence on the nutritional status. Anemia and hypoalbuminemia frequently occur in cancer patients and have a multifactorial etiogenesis reflecting both the nutritional and inflammatory status [36,37].

The involvement of inflammatory response elements such as neutrophils, lymphocytes [38], and platelets [39,40] in cancer immunosurveillance and immunoediting suggests an important role in the process of carcinogenesis and tumor development. Moreover, they may be responsible for the significantly decreased levels of WBC and NLR detected in the PDAC group. A decreased total lymphocyte count has been found in patients with PDAC and has been associated with a poor prognosis [41], while a recent meta-analysis showed how low NLR can be linked to longer survival in patients with advanced PDAC [42]. Hence, the negative prognostic role of NLR is confirmed in all stages of the disease; however, its role in the early detection of PDAC has not been clearly demonstrated [43]. No clinical parameter, alone or in combination with the others, was able to surpass the predictive capacity of corona analysis.

As a next step, we asked whether the outcomes of clinical biomarkers could improve the sensitivity and specificity of the corona analysis. When the hemoglobin levels and values of Area 2 from corona analysis were coupled, the global prediction ability increased to an impressive 90%. It is our hypothesis that modifications induced by the tumor may be responsible for protein profile modification in the range of 20–30 kDa detected in the PDAC group. This difference distinguished cancer patients from healthy subjects and supported what has already been reported about the ability of protein corona technology in detecting PDAC cases [44]. Notably, it has been proven that decreasing the WBC and lymphocytes resulted in the reduction in levels of cytokines such as interleukin 24 (molecular weight 23 kDa), which plays an important role in tumor immunosuppression [45]. Moreover, the reduction of other proteins such as IFN*γ* (molecular weight 21 kDa) contributed to the decrease in the number and activity of cells involved in immune surveillance. This decrease in immune surveillance exacerbated the impairment of the immune system by the tumor in PDAC cases [46]. This study has some limitations, the greatest of which is the small sample size analyzed. Moreover, the samples are confounded by the presence of all stages of the disease, with a higher prevalence of the most advanced stages. This is intrinsically related to the characteristic of the disease, which in most cases is identified at an advanced stage. If our findings are further confirmed in a broader cohort including early stage PDAC or in patients affected by chronic pancreatitis, corona analysis may pave the way to the development of novel detection technologies for PDAC meeting the ASSURED criteria. Moreover, beyond its role in cancer detection, protein corona characterization can also add value to alterations in blood tests parameters that are often detected in malignancies but that by themselves are burdened by very low specificity.

## 4. Materials and Methods

### 4.1. Patients’ Enrolment and Blood Sample Collection

The Ethical Committee of the University Campus Bio-Medico di Roma approved this study (Prot. 10/12 ComEt CBM). The study group comprised 34 PDAC (PDAC group) and 34 subjects (healthy controls) affected by benign surgical disease (e.g., cholelithiasis and/or groin hernia). All the participants were cytohistologically diagnosed and proven to be eligible. The inclusion criteria for the study were age ≥ 18 years; adequate renal function (creatinine < 1.5 mg/dL, blood urea nitrogen < 1.5 times the upper limit); previous personal medical history negative for neoplasticity; renal or liver disease or blood disorders; no previous chemotherapy or radiotherapy; absence of uncontrolled infections; and written, informed consent.

Demographic characteristics of PDAC patients and healthy subjects were reported in Table S1 of the Supplementary Materials (SM), while comorbidities were reported in Tables S2 and S3 of SM (respectively). PDAC patients had a median age of 73 years (range 47–86), 19 (56%) were male, and 15 (44%) were female. The diagnosis of PDAC was confirmed by radical surgery in 16 (47%) cases. Increased levels of CA19.9 were found in 28 (82%) cases. Distribution among stages according to the eight edition of TNM [47] was stage IA in 1 (4.2%), stage IB in 1 (4.2%), stage IIA in 1 (4.2%), stage IIB in 14 (56%), stage III (12%), and stage IV in 5 (20%) of patients, respectively.

The main comorbidities found were hypertension in 18 (53%), respiratory disease in 2 (6%), hypercholesterolemia in 2 (6%), diabetes mellitus in 3 (9%), hypothyroidism in 1 (3%), neurologic disorders in 3 (9%) and diverticular disease in 2 (6%), respectively.

Healthy controls had a median age of 55 (range 18–85); 19 (56%) were male and 15 (44%) were female. The baseline surgical pathologies were 18 (53%) cholelithiasis, 5 (15%) groin hernia, 3 (9%) haemorrhoids, 1 (3%) incisional hernia, 1 (3%) abdominal wall lipoma, 1 (3%) benign ovarian cyst, 1 (3%) chronic constipation, 3 (9%) diverticular disease, and 1 (3%) diaphragmatic hernia. Levels of CA19.9 were ≥37 UI/mL in 1 case (9%). The main comorbidities found were hypertension in 8 (24%), gastroesophageal reflux disease in 2 (6%), respiratory disease in 1 (3%), and hypercholesterolemia in 1 (3%), respectively.

Blood samples were obtained by a classic venepuncture technique and collected in TM BD P100 Blood Collection System (Franklin Lakes, NJ, USA) comprising test tubes with K2EDTA and a protease inhibitor solution. Plasma was obtained and stored according to procedures previously reported [48].

### 4.2. Preparation of Nanosized GO Sheets

Graphene Oxide was purchased from Donostia, Spain, (1 mg/mL, water dispersion) and sonicated for 30 min at 125 W by a vibra cell sonicator VC505 (Sonics and Materials, Newtown, UK) equipped with a 2 mm stepped microtip (Sonics and Materials, Part No. 630-0423). The GO solution was then centrifuged at 15,000× *g* for 100 min (Hermle Z 216 MK, Hermle Labortechnik). Lastly, the supernatant was recovered for subsequent use and its concentration (30 μg mL^−1^) was determined by UV-VIS experiments [49].

### 4.3. Size and Zeta-Potential Experiments

Solutions were characterized by dynamic light scattering (DLS) and electrophoretic light scattering (ELS) using a Zetasizer Nano ZS (Malvern, Herrenberg, Germany) instrument, equipped with a 633 nm He−Ne laser. Size measurement was performed with a non-invasive back scattering configuration (173°), at automatic position and attenuator, at room temperature. Zeta potential of GO was computed by measuring the samples’ electrophoretic mobility and taking into account the Henry correction to Smoluchowski’s equation. Results were reported as mean ± standard deviation of three replicates.

### 4.4. SDS-PAGE Experiments

GO flakes were exposed to 5% HP (*vol/vol*) collected from PDAC and controls. After a 1-h incubation at 37 °C, GO-protein complexes were isolated from excess plasma by a consolidated experimental procedure. Complexes were centrifuged at 15,000× *g* for 15 min at 4 °C. Pellets were washed tree times with cold PBS to remove unbound proteins. Then, pellets were resuspended with 40 μL of Leammli sample buffer 1× (supplemented with Sample reducing buffer 1×) and boiled at 100 °C for 10 min. Lastly, 10 μL of each sample was loaded on a Criterion TGX Stain-free precast gel (4–20% polyacrylamide gradient gels), which ran at 100 V for about 90 min. The gels were then washed in milliQ water and revealed using the Chemidoc MP System (Biorad, Hercules, CA, USA). Gel images were processed by custom Matlab (Mathwork, Natick, MA, USA) scripts to evaluate the one-dimensional intensity function of each sample and thus obtain the corresponding molecular weight distribution. All the experimental proceduresare well established and can be found elsewhere. [50,51]

### 4.5. ROC Analysis

Receiving operative curve (ROC) analysis and corresponding areas under curve (AUC) were calculated using Matlab (Mathwork).

## 5. Conclusions

Simultaneous detection of multiple analytes from a single sample and patient is gaining increasing relevance for the development of in vitro diagnostic devices, especially in resource-limited settings (e.g., countries in the developing world). Multiplexed technologies are providing new methodologies for cancer from a deeper understanding of the tumor microenvironment, to the development of new targets for treatment, and prognostic and predictive biomarkers. However, most of the developed technologies are expensive, and, in general, they do not meet the ASSURED criteria of the WHO. To address this gap, we developed a multiplexed strategy that combines values of clinical biomarkers with protein corona analysis. If further confirmed in larger prospective studies, this novel approach could potentially discriminate PDAC from healthy subjects in up to 90% of cases and be considered in the management of PDAC. Validation of the proposed approach in thousands of patients and mostly in early stage PDAC could facilitate its rapid translation into clinical practice as well as improve the specificity of alterations in common blood tests.

## Data Availability

The data presented in this study are available on request from the corresponding author.

## Supplementary Materials

The following are available online at https://www.mdpi.com/2072-6694/13/1/93/s1, Table S1: Demographic characteristics and serum markers of PDAC patients and healthy subjects. Table S2: Comorbidities and TNM stage according to AJCC 8th edition in PDAC patients. Table S3: Comorbidities and baseline surgical pathology in the healthy subjects. Figure S1: Global correctness of the test obtained by linear discriminant analysis for each possible couple of parameters. Figure S2: Scatter plot for the best couple of parameters.
