# Supplementary material for: Synergistic Analysis of Protein Corona and Haemoglobin Levels Detects Pancreatic Cancer
Source: Cancers (Basel). 2020 Dec 30;13(1):93. doi: 10.3390/cancers13010093 (PMC7796289; doi:10.3390/cancers13010093)
Supplement: Supplementary file 1 [file cancers-13-00093-s001.pdf]

Supplementary Material: Synergistic Analysis of Protein Corona and Haemoglobin Levels Detects Pancreatic Cancer

Damiano Caputo, Luca Digiacomo, Chiara Cascone, Daniela Pozzi, Sara Palchetti, Riccardo Di Santo, Erica Quagliarini, Roberto Coppola, Morteza Mahmoudi and Giulio Caracciolo

Table S1. Demographic characteristics and serum markers of PDAC patients and healthy subjects.

Characteristic	Criterion	PDAC patients (n = 34)	Healthy subjects (n = 34)
Age, y	Median (range)	73 (range 47–86)	55 (range 18–85)
Sex	Male, n (%)	19 (56%)	19 (56%)
	Female, n (%)	15 (44%)	15 (44%)
CA 19.9 (n.v. < 37 UI/mL)	< 37 UI/mL	6 (18%)	10 (91%)
	≥ 37 UI/mL	28 (82%)	1 (9%)

Table S2. Comorbidities and TNM stage according to AJCC 8th edition in PDAC patients.

Comorbidity	PDAC patients (n = 34)
Blood Hypertension	18 (53%)
Respiratory disease	2 (6%)
Hypercholesterolemia	2 (6%)
Diabetes mellitus	3 (9%)
Hypothyroidism	1 (3%)
Neurologic disorders	3 (9%)
Diverticular disease	2 (6%)
**TNM stage (AJCC 8th edition)**	
IA	1 (3%)
IB	3 (9%)
IIA	1 (3%)
IIB	18 (53%)
III	6 (18%)
IV	5 (15%)

Table S3. Comorbidities and baseline surgical pathology in the healthy subjects.

**Comorbidity**	**Healthy subjects (n = 34)**
Blood Hypertension	8 (24%)
Gastroesophageal Reflux Disease	2 (6%)
Respiratory disease	1 (3 %)
Hypercholesterolemia	1 (3%)
**Baseline surgical pathology**	
Cholelithiasis	18 (53%)
Inguinal hernia	5 (15%)
Hemorrhoids	3 (9%)
Incisional hernia	1 (3%)
Abdominal wall lipoma	1 (3%)
Ovarian cyst	1 (3%)
Chronic constipation	1 (3%)
Diverticular disease	3 (9%)
Diaphragmatic hermia	1 (3%)

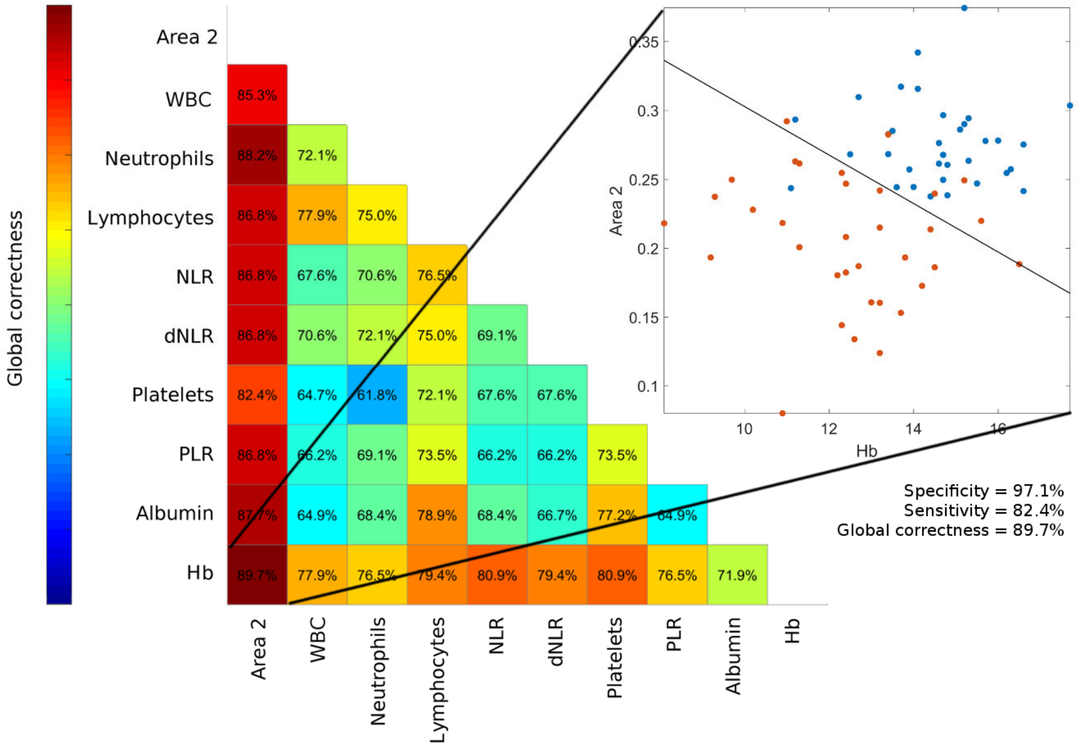

Figure S1. Global correctness of the test obtained by linear discriminant analysis for each possible couple of parameters. Scatter plot for the best coupling is represented in the inset. Each data point corresponds to a sample (orange for PDAC, blue for non-oncological coronas), solid black line depicts the output of the linear discriminant analysis for the two multivariate distributions.

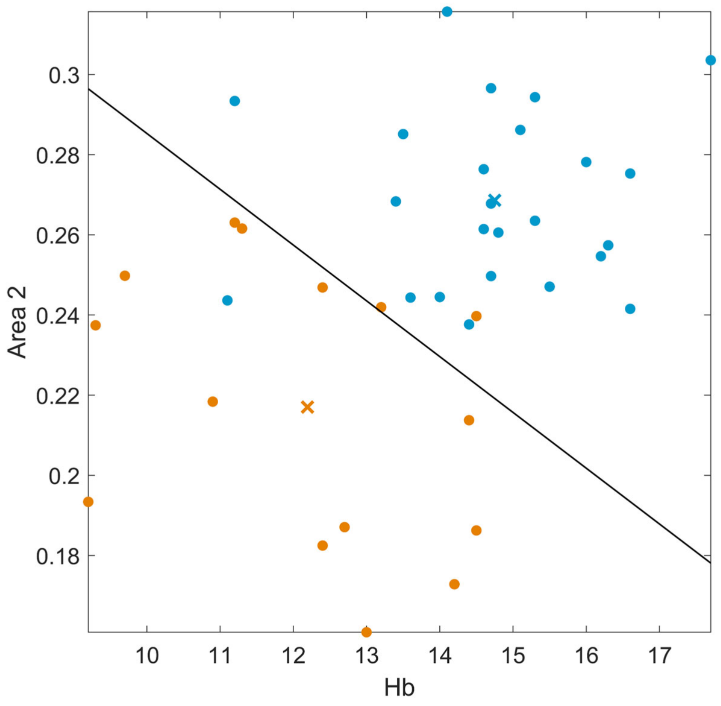

Figure S2. Scatter plot for the best couple of parameters, i.e. haemoglobin (Hb) concentration expressed in grams per decilitre and integral area underneath the 1D protein pattern retrieved by SDS-PAGE in the region of molecular weight between 20 and 30 kDa (Area 2). Orange points indicate PDAC patients, while blue points refer to healthy individuals. The solid black line depicts the output of the linear discriminant analysis for the two multivariate distributions.
